# A Bidirectional Mendelian Randomization Study of the Causal Association Between Ischemic Stroke, Coronary Heart Disease, and Hydrocephalus

**DOI:** 10.1002/brb3.70090

**Published:** 2024-10-08

**Authors:** Wencai Wang, Menghao Liu, Zun Wang, Luyao Ma, Yongqiang Zhao, Wei Ye, Xianfeng Li

**Affiliations:** ^1^ Department of Neurosurgery The Second Affiliated Hospital of Harbin Medical University Harbin People's Republic of China

**Keywords:** coronary heart disease, genetic, hydrocephalus, ischemic stroke, Mendelian randomization

## Abstract

**Background:**

The association among coronary heart disease, ischemic stroke, and hydrocephalus remains ambiguous.

**Objectives:**

There is a need for a Mendelian randomization study to evaluate the underlying causality between coronary heart disease, ischemic stroke, and hydrocephalus.

**Methods:**

The data source utilized genome‐wide association studies, employing a threshold of *p *< 5 × 10^−8^ to identify single nucleotide polymorphisms strongly linked to ischemic stroke and coronary heart disease as instrumental variables (IVs). Five methods—inverse variance weighted (IVW), Mendelian randomization (MR) Egger, Weighted Median, Weighted mode, and Simple mode—utilized the selected IVs to estimate the causality between ischemic stroke, coronary heart disease, and hydrocephalus.

**Results:**

The IVW demonstrated that ischemic stroke and coronary heart disease serve as risk factors for hydrocephalus (odds ratio [OR] = 1.650, 95% CI: 1.066–2.554, *p* = 0.025; OR = 1.307, 95% CI: 1.023–1.668, *p* = 0.032). Both the MR‐Egger intercept test and Cochran's *Q* test affirmed the relative reliability of the IVW analysis results. However, no evidence of a reverse causation was observed between hydrocephalus and coronary heart disease or ischemic stroke.

**Conclusions:**

Coronary heart disease and Ischemic stroke may increase the risk of hydrocephalus.

## Graphical abstract

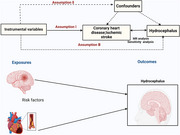

**Abbreviations**
CSF: cerebrospinal fluid
GWAS: genome‐wide association studies
IVs: instrumental variables
IVW: inverse variance weighted
LD: linkage disequilibrium
MR: Mendelian randomization
OR: odds ratio
RCTs: randomized controlled clinical trials
SNPs: single nucleotide polymorphisms
WM: weighted median

## 1 Introduction

Hydrocephalus, a serious neurosurgical condition, is characterized by an abnormal buildup of cerebrospinal fluid (CSF), which can lead to increased intracranial pressure. It is classified into congenital hydrocephalus, present at birth, and acquired hydrocephalus, which develops later in life. Therapeutic interventions encompass shunting procedures as well as endoscopic approaches (Kahle et al. 2016). It is a debilitating condition that affects individuals across all age groups and imposes a substantial financial burden on both patients and society (Isaacs et al. 2018). Thus, the identification and management of risk factors are crucial for the prevention of hydrocephalus. Cardiovascular and cerebrovascular diseases pose a significant threat to global public health and are also considered potential contributors to the development of hydrocephalus (Karakayali et al. 2023). A meta‐analysis of 25 observational studies highlighted ischemic heart disease as a significant risk factor for this condition (Walsh et al. 2017). Moreover, earlier observational studies have suggested an underlying association between hydrocephalus and cerebral infarction (Bakker et al. 2007; Masson et al. 2022).

Despite these findings, the exact association of these exposures with outcomes remains uncertain. Furthermore, observational studies have inherent limitations and may be susceptible to reverse causation and confounding factors, potentially impacting the accuracy of the results. Mendelian randomization (MR) analysis provides advantages similar to the randomization process in randomized controlled trials (RCTs), reducing the influence of confounding factors and remaining unaffected by reverse causality. Due to its simplicity and ease of implementation, MR has become a preferred approach when RCTs are not feasible (Thanassoulis and O'Donnell 2009). This study utilized MR analysis to explore the causality between ischemic stroke, coronary heart disease, and hydrocephalus, providing novel evidence to inform clinical interventions aimed at preventing hydrocephalus.

## 2 Methods

### 2.1 Study Design

**FIGURE 1:**
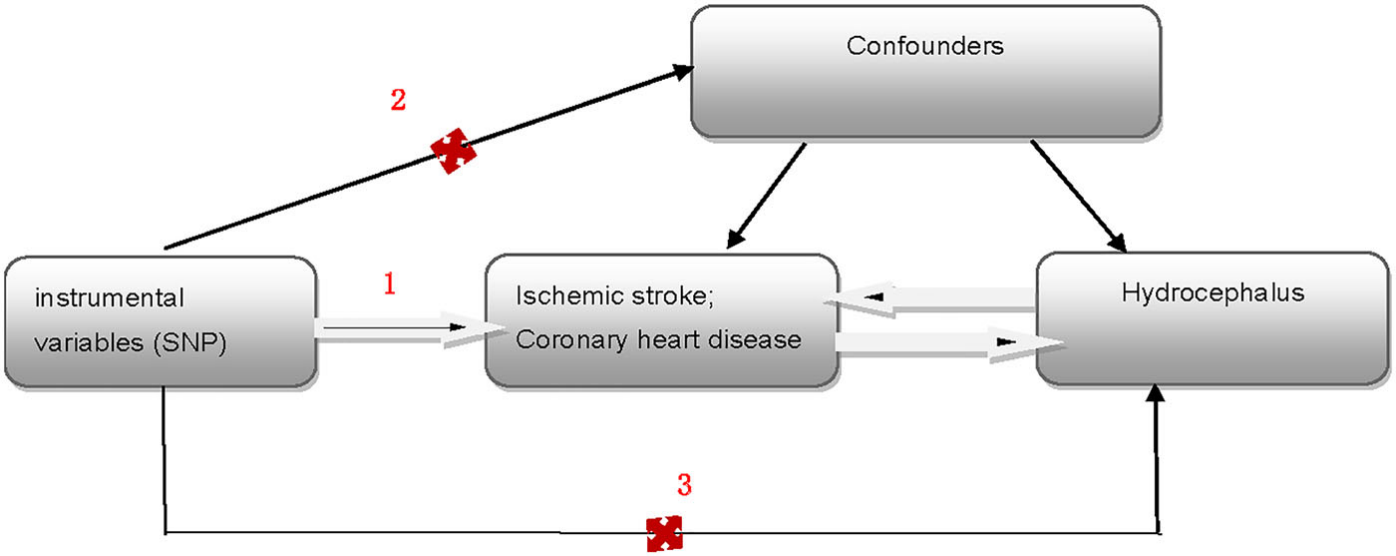
MR study of ischemic stroke, coronary heart disease, and hydrocephalus. MR, Mendelian randomization; SNP, single nucleotide polymorphism.

This article was prepared in accordance with the STROBE‐MR guidelines, and the analysis was rigorously conducted based on the three core assumptions of MR analysis (Skrivankova et al. 2021; Birney 2022): (1) The selected instrumental variables (IVs) are related to coronary heart disease and ischemic stroke; (2) the IVs are not confounded by external factors; and (3) the IVs influence the occurrence of hydrocephalus solely through ischemic stroke or coronary heart disease, respectively, and do not affect hydrocephalus via other pathways (Figure 1).

### 2.2 Genome‐Wide Association Studies (GWAS) Data Source

The GWAS data for ischemic stroke and coronary heart disease were sourced from the IEU database (https://gwas.mrcieu.ac.uk/), which included 34,217 ischemic stroke patients and 406,111 control subjects, with a total of 7537,579 SNPs, and 22,233 coronary heart disease patients and 64,762 control subjects, with a total of 2415,020 SNPs (Malik et al. 2018; Schunkert et al. 2011). The GWAS data for hydrocephalus were obtained from the Finnish database (https://r6.finngen.fi/), comprising 749 hydrocephalus patients and 205,799 control subjects, with a total of 16,380,404 SNPs. As our study utilized data from these original sources, additional ethical review and informed consent were not required.

### 2.3 Selection of IVs

We utilized a threshold of *p* < 5 × 10^−8^ to identify SNPs strongly associated with coronary heart disease and ischemic stroke from the GWAS summary dataset as IVs. Additionally, we applied a clumping distance of 10,000 kb and an *r* ^2^ threshold of 0.001 to select independent SNPs that are not affected by linkage disequilibrium (LD) (Sekula et al. 2016). To address the potential bias from weak IVs, SNPs with an *F*‐statistic of less than 10 were eliminated. Additionally, SNPs associated with confounding factors for ischemic stroke, coronary heart disease, and hydrocephalus were systematically excluded using the PhenoScanner database (http://www.phenoscanner.medschl.cam.ac.uk/).

### 2.4 Statistical Analysis

Statistical analysis was performed utilizing R 4.3.2 with the TwoSampleMR package (Hemani et al. 2018). The main analysis utilized the inverse‐variance weighted (IVW) method, with a significance threshold set at *p* < 0.05. Supplementary analyses included the weighted median (WM), MR‐Egger, weighted mode, and simple mode methods to ensure the stability and reliability of the results. The MR‐Egger regression intercept was used to test for horizontal pleiotropy, whereas the Cochrane *Q*‐test was employed to assess heterogeneity among the results (Bowden, Davey Smith, and Burgess 2015). A *p* value greater than 0.05 indicated the absence of pleiotropy or heterogeneity among the SNPs. The leave‐one‐out method was used to evaluate the influence of each SNP on the results individually, and MR‐PRESSO was employed to detect outliers. If outlier SNPs were identified, they were excluded and the analysis was re‐performed. All results were visualized using forest plots.

## 3 Results

### 3.1 Instrumental Variables

Eighteen SNPs were identified as IVs for assessing the causality between ischemic stroke and hydrocephalus (Table 1), whereas 14 SNPs were used for evaluating the causality between coronary heart disease and hydrocephalus (Table 2). All SNPs had an *F* statistic greater than 10, indicating robustness in evaluating the causal association between ischemic stroke and hydrocephalus (Table S1). The median *F* statistic for SNPs assessing the relationship between ischemic stroke and hydrocephalus was 38.87 (range: 29.57–66.69), and for coronary heart disease and hydrocephalus, it was 45.35 (range: 30.50–138.90).

**TABLE 1:** Assessing single nucleotide polymorphisms (SNPs) for causal association between ischemic stroke and hydrocephalus.

SNP	Chr	Effect_allele	Other_allele	Pval.exposure	Pval.outcome	Eaf.exposure	Eaf.outcome
rs1052053	1	G	A	4.48E − 11	0.200	0.401	0.345
rs1053007	19	G	A	3.58E − 08	0.767	0.651	0.717
rs11957829	5	G	A	7.51E − 09	0.775	0.176	0.197
rs12445022	16	A	G	1.28E − 10	0.829	0.306	0.319
rs17035646	1	A	G	1.34E − 09	0.409	0.405	0.397
rs2005108	11	T	C	3.33E − 08	0.260	0.128	0.183
rs2107595	7	A	G	9.25E − 14	0.465	0.226	0.194
rs3184504	12	C	T	2.17E − 14	0.107	0.548	0.592
rs35436	12	T	C	3.21E − 08	0.644	0.381	0.364
rs42039	7	T	C	6.55E − 09	0.403	0.228	0.233
rs4932370	15	A	G	2.88E − 08	0.423	0.333	0.274
rs4959130	6	A	G	2.83E − 09	0.173	0.137	0.166
rs6825454	4	C	T	7.43E − 10	0.139	0.308	0.317
rs6847935	4	T	A	3.50E − 16	0.314	0.326	0.237
rs7304841	12	C	A	4.93E − 08	0.204	0.407	0.404
rs7859727	9	T	C	1.05E − 09	0.086	0.536	0.414
rs9526212	13	G	A	9.19E − 10	0.720	0.761	0.761
rs9909858	17	C	T	3.63E − 08	0.826	0.188	0.051

**TABLE 2:** Assessing single nucleotide polymorphisms (SNPs) for causal association between coronary heart disease and hydrocephalus.

SNP	Chr	Effect_allele	Other_allele	Pval.exposure	Pval.outcome	Eaf.exposure	Eaf.outcome
rs10455872	6	G	A	3.08035E − 13	0.650	0.062	0.046
rs1122608	19	T	G	9.72994E − 10	0.771	0.235	0.213
rs11556924	7	T	C	2.21998E − 09	0.850	0.376	0.326
rs12190287	6	G	C	4.63981E − 11	0.257	0.377	0.448
rs1333045	9	C	T	4.6302E − 32	0.057	0.530	0.455
rs17114036	1	G	A	1.43001E − 08	0.640	0.089	0.109
rs2219939	15	A	G	1.21001E − 09	0.486	0.760	0.627
rs2306374	3	C	T	3.34003E − 08	0.321	0.180	0.107
rs2351524	2	C	T	1.75995E − 11	0.317	0.877	0.887
rs4714955	6	T	C	6.02976E − 12	0.778	0.356	0.285
rs599839	1	A	G	2.89001E − 10	0.347	0.777	0.780
rs7651039	3	C	T	1.84999E − 08	0.571	0.543	0.507
rs9351814	6	C	A	2.01999E − 08	0.444	0.381	0.389
rs964184	11	C	G	8.01992E − 10	0.591	0.868	0.854
rs9982601	21	T	C	4.21998E − 10	0.559	0.151	0.137

### 3.2 MR Analysis Results

**FIGURE 2:**
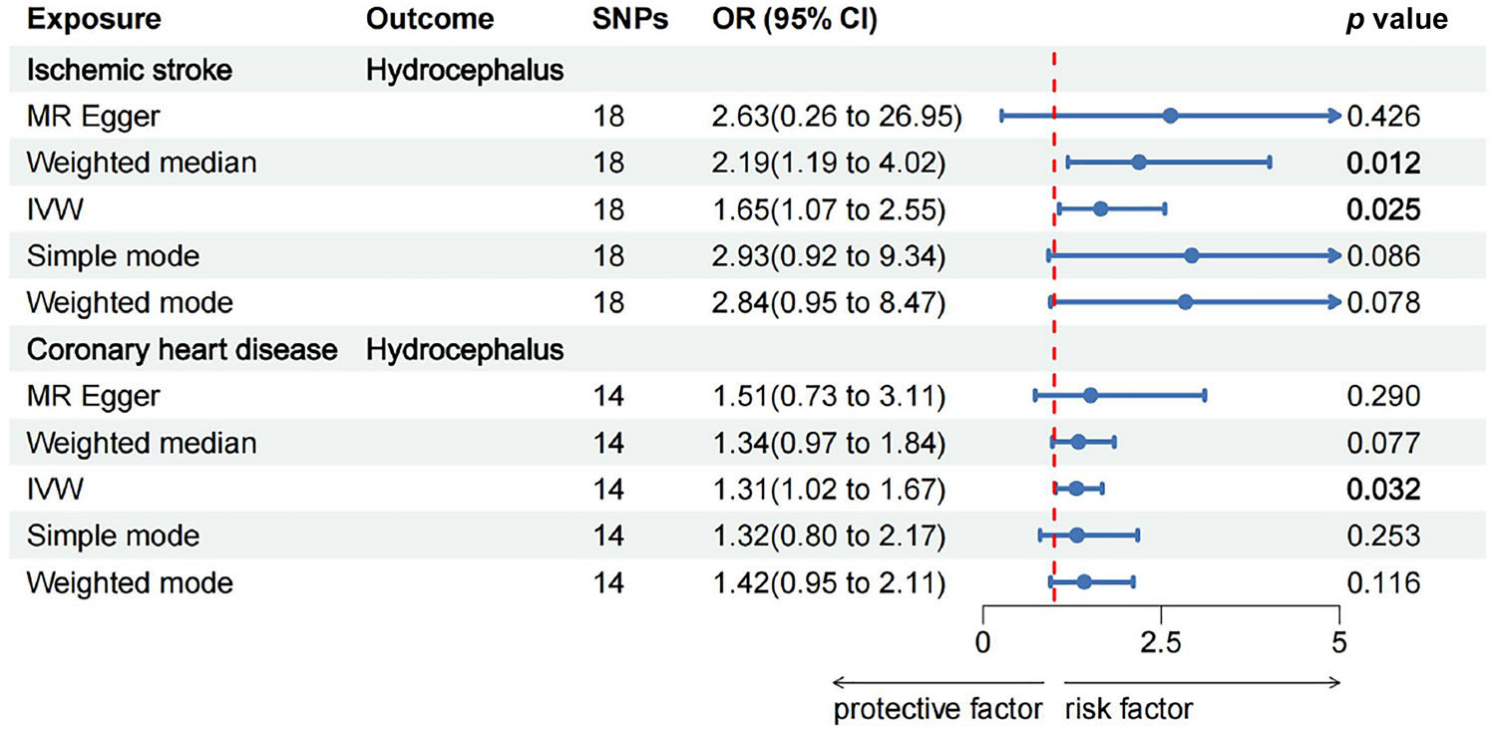
Forest plot for MR estimates from genetically predicted ischemic stroke, coronary heart disease effect on hydrocephalus. MR, Mendelian randomization; SNP, single nucleotide polymorphism.

**FIGURE 3:**
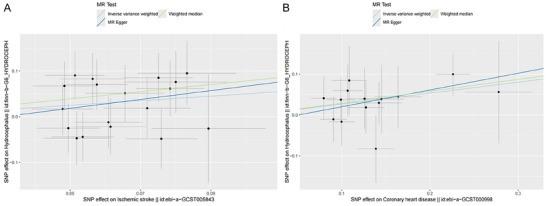
Scatter plots of MR estimates from genetically predicted ischemic stroke (A), coronary heart disease (B) effect on hydrocephalus. IVW, inverse variance weighted; MR, Mendelian randomization; OR, odds ratio; SNP, single nucleotide polymorphism.

The MR analysis results suggested a causal relationship between ischemic stroke, coronary heart disease, and hydrocephalus, as illustrated in Figure 2. Figure 3 depicts the relationship between SNPs, exposure, and outcome. The findings revealed that ischemic stroke is a risk factor for hydrocephalus, with the WM method yielding an odds ratio (OR) of 2.19 (95% CI: 1.19–4.02) and the IVW method yielding an OR of 1.65 (95% CI: 1.07–2.55), both statistically significant with *p* < 0.05. Specifically, the IVW analysis indicated a 65% increase in the risk of hydrocephalus for every 1 unit increase in ischemic stroke. Additionally, the IVW analysis showed that coronary heart disease is also a risk factor for hydrocephalus, with an OR of 1.31 (95% CI: 1.02–1.67), *p* < 0.05. For every 1 unit increase in coronary heart disease, the risk of hydrocephalus rises by 31%, according to the IVW analysis. However, reverse MR did not show evidence of reverse causation between hydrocephalus and ischemic stroke or coronary heart disease (Table S2).

### 3.3 Sensitivity Analysis of the Results

The MR‐Egger intercept test results indicated that the Egger‐intercept (beta) values for the MR estimations of ischemic stroke and hydrocephalus were − 0.030 (*p* = 0.693), and for coronary heart disease and hydrocephalus, it was − 0.021 (*p* = 0.690). These findings suggested that genetic polymorphisms are unlikely to influence the MR analysis results in this study. Sensitivity analysis using the leave‐one‐out method confirms the robustness of the MR results (Supporting Information Figure). Additionally, both MR‐Egger and IVW methodologies yielded Cochran's *Q*‐test *p* values greater than 0.05, indicating no heterogeneity in the MR analysis results for ischemic stroke, coronary heart disease, and hydrocephalus (Table 3).

**TABLE 3:** Mendelian randomization (MR) sensitivity analysis results.

Exposures	Analytical method	*Q*	*Q*_pval	Egger_intercept_P	MR‐PRESSO_P
Ischemic stroke	MR Egger	13.07	0.668		
	IVW	13.23	0.721	0.693	0.737
Coronary heart disease	MR Egger	4.39	0.975		
	IVW	4.56	0.984	0.690	0.983

Abbreviations: IVW, inverse variance weighted; MR, Mendelian randomization.

## 4 Discussion

This research employed MR analysis to investigate the causal relationships between ischemic stroke, coronary heart disease, and hydrocephalus. The findings revealed that both ischemic stroke and coronary heart disease are risk factors for hydrocephalus. Specifically, each unit increase in ischemic stroke is associated with a 65% increase in the risk of hydrocephalus, whereas each unit increase in coronary heart disease corresponds to a 31% increase in the risk of hydrocephalus.

Numerous prior studies have demonstrated a strong association between vascular risk factors and the progression of hydrocephalus (Cai et al. 2023; Israelsson et al. 2017). Although hemorrhagic stroke has been well‐established as a significant cause of secondary hydrocephalus, the relationship between ischemic stroke and hydrocephalus remains unclear (Sadegh et al. 2023). Our study confirms that ischemic stroke is indeed a risk factor for hydrocephalus. Ischemic stroke, characterized by interruptions in brain blood supply leading to localized ischemia, hypoxic necrosis, and subsequent neurological deficits, is a clinical syndrome with a multifactorial etiology (Walter 2022). It is often associated with chronic inflammation, disruption of the blood–brain barrier, and accumulation of toxic metabolites. This condition manifests as impaired blood flow and metabolism in the deep white matter, reduced periventricular metabolism, and decreased cerebral blood flow in the periventricular and frontal subcortical regions (Feske 2021; Qiu et al. 2021; Kuriakose and Xiao 2020). Furthermore, ischemic stroke is a common macrovascular disorder in which central arterial sclerosis leads to a chronic increase in cerebral artery pulsation, indirectly heightening CSF pulsation. Molecular abnormalities involved in the pathogenesis of hydrocephalus include disruptions in brain development, ependymal cell dysfunction, apoptosis, inflammation, free radical generation, blood flow, and brain metabolism. The pathophysiology often involves hypoxia and vascular changes (Li et al. 2021; Chahlavi, El‐Babaa, and Luciano 2001). During an ischemic stroke, the occlusion of small arteries and associated veins worsens CSF absorption impairment, leading to increased ventricular dilation and damage to pericerebral perfusion. Concurrently, there is a loss of the brain's autoregulatory function and ischemic changes in the deep white matter. Additionally, reduced periventricular metabolism due to ischemia and hypoxia results in toxin accumulation, which further exacerbates the development of hydrocephalus (Edwards et al. 2004; Yamada, Ishikawa, and Nozaki 2021). The prognosis of hydrocephalus is closely related to the etiology and severity of the stroke. Effective management often requires considering these factors to improve patient outcomes.

Coronary heart disease is the most common form of ischemic heart disease, and numerous observational studies have shown an association between coronary heart disease and hydrocephalus (Jaraj et al. 2016; Casmiro et al. 1989; Krauss et al. 1996; Román et al. 2018). A meta‐analysis of 11 case–control studies supported our MR findings, indicating that coronary heart disease increases the risk of hydrocephalus (Cai et al. 2023). Coronary heart disease is a cardiovascular condition characterized by the narrowing or blockage of the coronary arteries, which leads to an inadequate blood supply to the heart (Karakayali et al. 2023; Klocke et al. 2007). Coronary heart disease affects the heart's ability to pump blood effectively, which can lead to reduced blood supply to the brain (Kelley and Kelley 2021). As a result, oxygen deprivation in the brain can impact both the production and absorption of CSF. Additionally, coronary heart disease may be associated with vascular sclerosis and atherosclerosis, which can impair the function of the brain's blood vessels and CSF system, thereby disrupting CSF flow and absorption (Mera 1993). Furthermore, coronary heart disease can trigger various cerebrovascular disorders, which may subsequently disrupt the flow and absorption of CSF (Kovacic, Castellano, and Fuster 2012; Nicholls and Johansen 1983). Coronary heart disease is primarily atherosclerotic, causing ischemic and hypoxic damage to cerebral vessels and brain tissue. This results in significant alterations in metabolism, blood–brain barrier function, and CSF dynamics, which can lead to demyelination and ventricular enlargement in affected patients (Tsao et al. 2022). This cascade of pathological changes in the brain may underlie the development of hydrocephalus. This cascade of pathological alterations in the brain may serve as the underlying pathophysiological mechanism of hydrocephalus.

The strengths of this study include the following: (1) Observational studies are often affected by confounding factors and reverse causality, which can skew results. In contrast, the MR method utilizes large GWAS datasets to make more reliable causal inferences and reduce the bias commonly seen in observational studies; (2) although RCTs are expensive and difficult to conduct, MR studies can simulate RCTs to assess causality more feasibly; (3) our MR analysis examined the relationships between ischemic stroke, coronary heart disease, and hydrocephalus, revealing potential increased risks of hydrocephalus associated with coronary heart disease and ischemic stroke.

Nonetheless, the study has several limitations. First, the use of genetic variants may affect the validity of the MR analysis, as there is a risk of violating the typical assumptions of IVs, which could potentially distort the results. However, sensitivity analyses showed no evidence of such violations. Second, although the IVW and WM analyses indicated a positive causal relationship between ischemic stroke and hydrocephalus, the MR‐Egger results did not corroborate these findings. This discrepancy may arise from MR‐Egger's assumption that all SNPs are invalid IVs, which reduces its statistical power (Bowden, Davey Smith, and Burgess 2015). Nevertheless, the direction of the MR‐Egger estimate is consistent with that of the other MR methods. Overall, we remain confident in the positive causal relationship based on the robustness of the other analyses. Third, as the GWAS data are all derived from European populations, it remains unclear whether our results can be generalized to other ethnic groups.

## 5 Conclusion

These MR analyses support a causal effect of coronary heart disease and ischemic stroke on hydrocephalus, but not the other way around, indicating that ischemic stroke and coronary heart disease may increase the risk of developing hydrocephalus. In the future, greater attention may be needed to address the potential risk of hydrocephalus when treating coronary heart disease and ischemic stroke, with a particular focus on early screening and preventive measures for high‐risk groups.

## Author Contributions

**Wencai Wang**: conceptualization, investigation, writing–original draft, methodology, writing–review and editing, visualization, validation, software, formal analysis, data curation, supervision, resources, project administration. **Menghao Liu**: conceptualization, visualization, writing–review and editing. **Zun Wang**: conceptualization, methodology. **Luyao Ma**: conceptualization. **Yongqiang Zhao**: conceptualization. **Wei Ye**: conceptualization. **Xianfeng Li**: conceptualization, funding acquisition, project administration, writing–review and editing.

## Ethics Statement

The authors have nothing to report.

## Conflicts of Interest

The authors declare no conflicts of interest.

### Peer Review

The peer review history for this article is available at https://publons.com/publon/10.1002/brb3.70090.

## Supporting information



Additional supporting information can be found online in the Supporting Information section.

Additional supporting information can be found online in the Supporting Information section.

Additional supporting information can be found online in the Supporting Information section.

## Data Availability

The datasets analyzed during the current study are available in the IEU database, https://gwas.mrcieu.ac.uk/, and the FinnGen repository, https://r6.finngen.fi/.

## References

[brb370090-bib-0001] Bakker, A. M. , S. M. Dorhout Mees , A. Algra , and G. J. Rinkel . 2007. “Extent of Acute Hydrocephalus After Aneurysmal Subarachnoid Hemorrhage as a Risk Factor for Delayed Cerebral Infarction.” Stroke; A Journal of Cerebral Circulation 38, no. 9: 2496–2499. 10.1161/STROKEAHA.107.484220. 17673710

[brb370090-bib-0002] Birney, E. 2022. “Mendelian Randomization.” Cold Spring Harbor Perspectives in Medicine 12, no. 4: a041302. 34872952 10.1101/cshperspect.a041302 PMC9121891

[brb370090-bib-0003] Bowden, J. , G. Davey Smith , and S. Burgess . 2015. “Mendelian Randomization With Invalid Instruments: Effect Estimation and Bias Detection Through Egger Regression.” International Journal of Epidemiology 44, no. 2: 512–525. 10.1093/ije/dyv080. 26050253 PMC4469799

[brb370090-bib-0004] Cai, H. , F. Yang , H. Gao , et al. 2023. “Vascular Risk Factors for Idiopathic Normal Pressure Hydrocephalus: A Systematic Review and Meta‐Analysis.” Frontiers in Neurology 14: 1220473. 10.3389/fneur.2023.1220473. 37638192 PMC10448702

[brb370090-bib-0005] Casmiro, M. , R. D'Alessandro , F. M. Cacciatore , R. Daidone , F. Calbucci , and E. Lugaresi . 1989. “Risk Factors for the Syndrome of Ventricular Enlargement With Gait Apraxia (Idiopathic Normal Pressure Hydrocephalus): A Case‐Control Study.” Journal of Neurology, Neurosurgery, and Psychiatry 52, no. 7: 847–852. 10.1136/jnnp.52.7.847. 2769278 PMC1031931

[brb370090-bib-0006] Chahlavi, A. , S. K. El‐Babaa , and M. G. Luciano . 2001. “Adult‐Onset Hydrocephalus.” Neurosurgery Clinics of North America 12, no. 4: 753–760. 10.1016/S1042-3680(18)30032-9. 11524296

[brb370090-bib-0007] Edwards, R. J. , S. M. Dombrowski , M. G. Luciano , and I. K. Pople . 2004. “Chronic Hydrocephalus in Adults.” Brain Pathology 14, no. 3: 325–336. 10.1111/j.1750-3639.2004.tb00072.x. 15446589 PMC8096062

[brb370090-bib-0008] Feske, S. K. 2021. “Ischemic Stroke.” American Journal of Medicine 134, no. 12: 1457–1464. 10.1016/j.amjmed.2021.07.027. 34454905

[brb370090-bib-0009] Hemani, G. , J. Zheng , B. Elsworth , et al. 2018. “The MR‐Base Platform Supports Systematic Causal Inference Across the human Phenome.” eLife 7: e34408. 10.7554/eLife.34408. 29846171 PMC5976434

[brb370090-bib-0010] Isaacs, A. M. , J. Riva‐Cambrin , D. Yavin , et al. 2018. “Age‐Specific Global Epidemiology of Hydrocephalus: Systematic Review, Metanalysis and Global Birth Surveillance.” PLoS ONE 13, no. 10: e0204926. 10.1371/journal.pone.0204926. 30273390 PMC6166961

[brb370090-bib-0011] Israelsson, H. , B. Carlberg , C. Wikkelsö , et al. 2017. “Vascular Risk Factors in INPH: A Prospective Case‐Control Study (the INPH‐CRasH Study).” Neurology 88, no. 6: 577–585. 10.1212/WNL.0000000000003583. 28062721 PMC5304464

[brb370090-bib-0012] Jaraj, D. , S. Agerskov , K. Rabiei , et al. 2016. “Vascular Factors in Suspected Normal Pressure Hydrocephalus: A Population‐Based Study.” Neurology 86, no. 7: 592–599. 10.1212/WNL.0000000000002369. 26773072 PMC4762415

[brb370090-bib-0013] Kahle, K. T. , A. V. Kulkarni , D. D. Limbrick Jr. , and B. C. Warf . 2016. “Hydrocephalus in Children.” Lancet 387, no. 10020: 788–799. 10.1016/S0140-6736(15)60694-8. 26256071

[brb370090-bib-0014] Karakayali, M. , T. Omar , I. Artac , et al. 2023. “The Prognostic Value of HALP Score in Predicting in‐Hospital Mortality in Patients With ST‐Elevation Myocardial Infarction Undergoing Primary Percutaneous Coronary Intervention.” Coronary Artery Disease 34, no. 7: 483–488. 10.1097/MCA.0000000000001271. 37799045

[brb370090-bib-0015] Kelley, R. E. , and B. P. Kelley . 2021. “Heart‐Brain Relationship in Stroke.” Biomedicines 9, no. 12: 1835. 10.3390/biomedicines9121835. 34944651 PMC8698726

[brb370090-bib-0016] Klocke, R. , W. Tian , M. T. Kuhlmann , and S. Nikol . 2007. “Surgical Animal Models of Heart Failure Related to Coronary Heart Disease.” Cardiovascular Research 74, no. 1: 29–38. 10.1016/j.cardiores.2006.11.026. 17188668

[brb370090-bib-0017] Kovacic, J. C. , J. M. Castellano , and V. Fuster . 2012. “The Links Between Complex Coronary Disease, Cerebrovascular Disease, and Degenerative Brain Disease.” Annals of the New York Academy of Sciences 1254: 99–105. 10.1111/j.1749-6632.2012.06482.x. 22548575

[brb370090-bib-0018] Krauss, J. K. , J. P. Regel , W. Vach , D. W. Droste , J. J. Borremans , and T. Mergner . 1996. “Vascular Risk Factors and Arteriosclerotic Disease in Idiopathic Normal‐Pressure Hydrocephalus of the Elderly.” Stroke; A Journal of Cerebral Circulation 27, no. 1: 24–29. 10.1161/01.STR.27.1.24. 8553398

[brb370090-bib-0019] Kuriakose, D. , and Z. Xiao . 2020. “Pathophysiology and Treatment of Stroke: Present Status and Future Perspectives.” International Journal of Molecular Sciences 21, no. 20: 7609. 10.3390/ijms21207609. 33076218 PMC7589849

[brb370090-bib-0020] Li, J. , X. Zhang , J. Guo , C. Yu , and J. Yang . 2021. “Molecular Mechanisms and Risk Factors for the Pathogenesis of Hydrocephalus.” Frontiers in Genetics 12: 777926. 10.3389/fgene.2021.777926. 35047005 PMC8762052

[brb370090-bib-0021] Malik, R. , G. Chauhan , M. Traylor , et al. 2018. “Multiancestry Genome‐Wide Association Study of 520,000 Subjects Identifies 32 Loci Associated With Stroke and Stroke Subtypes.” Nature Genetics 50, no. 4: 524–537. 10.1038/s41588-018-0058-3. 29531354 PMC5968830

[brb370090-bib-0022] Masson, A. , G. Boulouis , K. Janot , et al. 2022. “Acute Hydrocephalus and Delayed Cerebral Infarction After Aneurysmal Subarachnoid Hemorrhage.” Acta Neurochirurgica 164, no. 9: 2401–2408. 10.1007/s00701-022-05321-8. 35918615

[brb370090-bib-0023] Mera, S. L. 1993. “Atherosclerosis and Coronary Heart Disease.” British Journal of Biomedical Science 50, no. 3: 235–248. 8241839

[brb370090-bib-0024] Nicholls, E. S. , and H. L. Johansen . 1983. “Implications of Changing Trends in Cerebrovascular and Ischemic Heart Disease Mortality.” Stroke; A Journal of Cerebral Circulation 14, no. 2: 153–156. 10.1161/01.STR.14.2.153. 6836641

[brb370090-bib-0025] Qiu, Y.‐M. , C.‐L. Zhang , A.‐Q. Chen , et al. 2021. “Immune Cells in the BBB Disruption after Acute Ischemic Stroke: Targets for Immune Therapy?” Frontiers in Immunology 12: 678744. 10.3389/fimmu.2021.678744. 34248961 PMC8260997

[brb370090-bib-0026] Román, G. C. , A. K. Verma , Y. J. Zhang , and S. H. Fung . 2018. “Idiopathic Normal‐Pressure Hydrocephalus and Obstructive Sleep Apnea Are Frequently Associated: A Prospective Cohort Study.” Journal of the Neurological Sciences 395: 164–168. 10.1016/j.jns.2018.10.005. 30340088

[brb370090-bib-0027] Sadegh, C. , H. Xu , J. Sutin , et al. 2023. “Choroid Plexus‐Targeted NKCC1 Overexpression to Treat Post‐Hemorrhagic Hydrocephalus.” Neuron 111, no. 10: 1591–1608.e4. 10.1016/j.neuron.2023.02.020. 36893755 PMC10198810

[brb370090-bib-0028] Schunkert, H. , I. R. König , S. Kathiresan , et al. 2011. “Large‐Scale Association Analysis Identifies 13 New Susceptibility Loci for Coronary Artery Disease.” Nature Genetics 43, no. 4: 333–338. 10.1038/ng.784. 21378990 PMC3119261

[brb370090-bib-0029] Sekula, P. , F. Del Greco M , C. Pattaro , and A. Köttgen . 2016. “Mendelian Randomization as an Approach to Assess Causality Using Observational Data.” Journal of the American Society of Nephrology 27, no. 11: 3253–3265. 10.1681/ASN.2016010098. 27486138 PMC5084898

[brb370090-bib-0030] Skrivankova, V. W. , R. C. Richmond , B. A. R. Woolf , et al. 2021. “Strengthening the Reporting of Observational Studies in Epidemiology Using Mendelian Randomization: The STROBE‐MR Statement.” JAMA 326, no. 16: 1614–1621. 10.1001/jama.2021.18236. 34698778

[brb370090-bib-0031] Thanassoulis, G. , and C. J. O'donnell . 2009. “Mendelian Randomization: Nature's Randomized Trial in the Post‐Genome Era.” JAMA 301, no. 22: 2386–2388. 10.1001/jama.2009.812. 19509388 PMC3457799

[brb370090-bib-0032] Tsao, C. W. , A. W. Aday , Z. I. Almarzooq , et al. 2022. “Heart Disease and Stroke Statistics‐2022 Update: A Report From the American Heart Association.” Circulation 145, no. 8: e153–e639. 35078371 10.1161/CIR.0000000000001052

[brb370090-bib-0033] Walsh, S. , J. Donnan , A. Morrissey , et al. 2017. “A Systematic Review of the Risks Factors Associated With the Onset and Natural Progression of Hydrocephalus.” Neurotoxicology 61: 33–45. 10.1016/j.neuro.2016.03.012. 27000516

[brb370090-bib-0034] Walter, K. 2022. “What Is Acute Ischemic Stroke?” JAMA 327, no. 9: 885. 10.1001/jama.2022.1420. 35230392

[brb370090-bib-0035] Yamada, S. , M. Ishikawa , and K. Nozaki . 2021. “Exploring Mechanisms of Ventricular Enlargement in Idiopathic Normal Pressure Hydrocephalus: A Role of Cerebrospinal Fluid Dynamics and Motile Cilia.” Fluids Barriers CNS 18, no. 1: 20. 10.1186/s12987-021-00243-6. 33874972 PMC8056523

