# Supplementary material for: A Bidirectional Mendelian Randomization Study of the Causal Association Between Ischemic Stroke, Coronary Heart Disease, and Hydrocephalus
Source: Brain Behav. 2024 Oct 8;14(10):e70090. doi: 10.1002/brb3.70090 (PMC11460635; doi:10.1002/brb3.70090)
Supplement: Supplementary file 1 — Additional supporting information can be found online in the Supporting Information section. [file BRB3-14-e70090-s002.docx]

**Supplement Figure: funnel plots, forest plots and leave-one-out plots for MR estimates from genetically predicted ischemic stroke, coronary heart disease effect on hydrocephalus.**

**
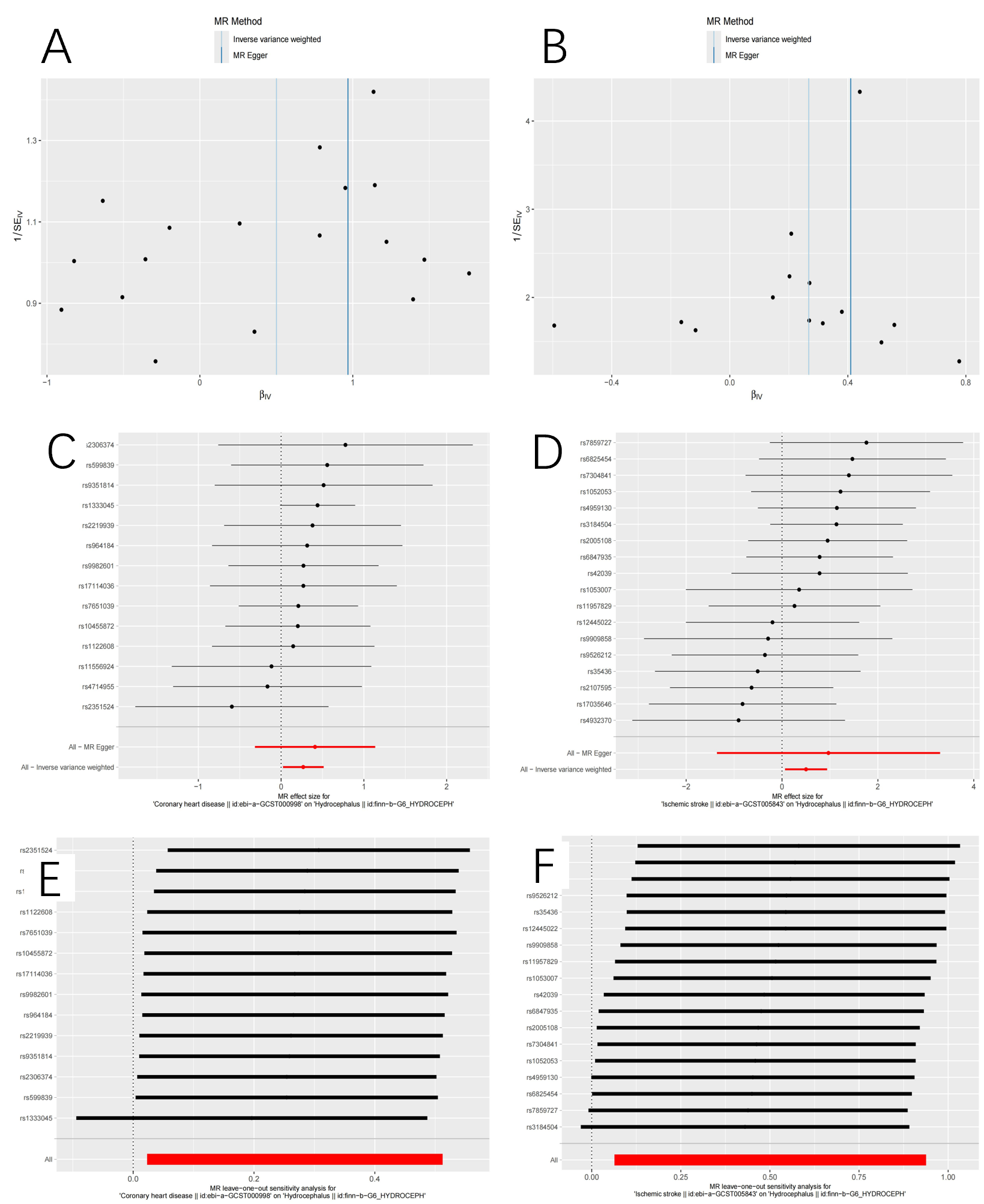
**

**A funnel plot for MR estimates from genetically predicted ischemic stroke effect on hydrocephalus.**

**B funnel plot for MR estimates from genetically predicted coronary heart disease effect on hydrocephalus.**

**C forest plot for MR estimates from genetically predicted ischemic stroke effect on hydrocephalus.**

**D forest plot for MR estimates from genetically predicted coronary heart disease effect on hydrocephalus.**

**E leave-one-out plot for MR estimates from genetically predicted ischemic stroke effect on hydrocephalus.**

**F leave-one-out plot for MR estimates from genetically predicted coronary heart disease effect on hydrocephalus.**
