# Supplementary material for: A Bidirectional Mendelian Randomization Study of the Causal Association Between Ischemic Stroke, Coronary Heart Disease, and Hydrocephalus
Source: Brain Behav. 2024 Oct 8;14(10):e70090. doi: 10.1002/brb3.70090 (PMC11460635; doi:10.1002/brb3.70090)
Supplement: Supplementary file 2 — Additional supporting information can be found online in the Supporting Information section. [file BRB3-14-e70090-s003.docx]

**Supplement Table 1：F value of SNPs strongly related to ischemic stroke and coronary heart disease.**

| **SNP (ischemic stroke)** | **F (ischemic stroke）** | **SNP (coronary heart disease)** | | **F (coronary heart disease)** |
| --- | --- | --- | --- | --- |
| **rs1052053** | **43.83353** | **rs17114036** | **32.14493** | |
| **rs17035646** | **37.09917** | **rs599839** | **39.75094** | |
| **rs6825454** | **37.58223** | **rs2351524** | **45.22628** | |
| **rs6847935** | **66.69444** | **rs2306374** | **30.49755** | |
| **rs11957829** | **33.62129** | **rs7651039** | **31.64689** | |
| **rs4959130** | **35.31755** | **rs4714955** | **47.3214** | |
| **rs2107595** | **55.37111** | **rs10455872** | **53.15692** | |
| **rs42039** | **33.70162** | **rs9351814** | **31.47071** | |
| **rs7859727** | **37.44274** | **rs12190287** | **43.32158** | |
| **rs2005108** | **30.43995** | **rs11556924** | **35.7742** | |
| **rs3184504** | **58.72564** | **rs1333045** | **138.9014** | |
| **rs35436** | **30.93359** | **rs964184** | **37.75685** | |
| **rs7304841** | **29.57404** | **rs2219939** | **36.95943** | |
| **rs9526212** | **37.07725** | **rs1122608** | **37.37777** | |
| **rs4932370** | **30.4845** | **rs9982601** | **39.00944** | |
| **rs12445022** | **41.09485** |  |  | |
| **rs9909858** | **30.38595** |  |  | |
| **rs1053007** | **30.31325** |  |  | |
