# Supplementary material for: A Bidirectional Mendelian Randomization Study of the Causal Association Between Ischemic Stroke, Coronary Heart Disease, and Hydrocephalus
Source: Brain Behav. 2024 Oct 8;14(10):e70090. doi: 10.1002/brb3.70090 (PMC11460635; doi:10.1002/brb3.70090)
Supplement: Supplementary file 3 — Additional supporting information can be found online in the Supporting Information section. [file BRB3-14-e70090-s001.docx]

Supplement Table 2：Main MR results of ischemic stroke, coronary heart disease and hydrocephalus.

| **Exposure** | **Outcome** | **SNPs** | **OR（95%Cl）** | **P-value** | **Q** | **Q_pval** | **egger_intercept_P** | **MR-PRESSO_P** |
| --- | --- | --- | --- | --- | --- | --- | --- | --- |
| **Ischemic stroke** | **Hydrocephalus** |  |  |  |  |  |  |  |
| MR Egger |  | 18 | 2.63 (0.26,26.95) | 0.426 | 13.07 | 0.668 |  |  |
| Weighted median |  | 18 | 2.19 (1.19,4.02) | 0.012 |  |  |  |  |
| IVW |  | 18 | 1.65 (1.07,2.55) | 0.025 | 13.23 | 0.721 | 0.693 | 0.737 |
| Simple mode |  | 18 | 2.93 (0.92,9.34) | 0.086 |  |  |  |  |
| Weighted mode |  | 18 | 2.84 (0.95,8.47) | 0.078 |  |  |  |  |
| **Coronary heart disease** | **Hydrocephalus** |  |  |  |  |  |  |  |
| MR Egger |  | 14 | 1.51 (0.73,3.11) | 0.290 | 4.39 | 0.975 |  |  |
| Weighted median |  | 14 | 1.34 (0.97,1.84) | 0.077 |  |  |  |  |
| IVW |  | 14 | 1.31 (1.02,1.67) | 0.032 | 4.56 | 0.984 | 0.690 | 0.983 |
| Simple mode |  | 14 | 1.32 (0.80,2.17) | 0.253 |  |  |  |  |
| Weighted mode |  | 14 | 1.42 (0.95,2.11) | 0.116 |  |  |  |  |
| **Hydrocephalus** | **Coronary heart disease** |  |  |  |  |  |  |  |
| MR Egger |  | 4 | 0.89 (0.48,1.67) | 0.755 | 1.83 | 0.402 |  |  |
| Weighted median |  | 4 | 0.95 (0.89,1.02) | 0.171 |  |  |  |  |
| IVW |  | 4 | 0.97 (0.92,1.03) | 0.349 | 1.90 | 0.594 | 0.812 | 0.612 |
| Simple mode |  | 4 | 0.95 (0.86,1.04) | 0.353 |  |  |  |  |
| Weighted mode |  | 4 | 0.95 (0.85,1.05) | 0.387 |  |  |  |  |
| **Hydrocephalus** | **Ischemic stroke** |  |  |  |  |  |  |  |
| MR Egger |  | 6 | 1.05 (0.91,1.22) | 0.529 | 7.32 | 0.120 |  |  |
| Weighted median |  | 6 | 1.02 (0.98,1.06) | 0.350 |  |  |  |  |
| IVW |  | 6 | 1.01 (0.97,1.04) | 0.624 | 7.96 | 0.159 | 0.585 | 0.185 |
| Simple mode |  | 6 | 1.03 (0.98,1.08) | 0.341 |  |  |  |  |
| Weighted mode |  | 6 | 1.03 (0.97,1.08 | 0.420 |  |  |  |  |
